# A mixed-methods evaluation of the one-way door and CitySafe patrol policies in Whangarei, New Zealand

**DOI:** 10.1371/journal.pone.0270149

**Published:** 2022-06-21

**Authors:** Michael P. Cameron, Juliana Brown, William Cochrane, Neville Robertson

**Affiliations:** 1 School of Accounting, Finance and Economics, University of Waikato, Hamilton, New Zealand; 2 Te Ngira – Institute for Population Research, University of Waikato, Hamilton, New Zealand; 3 School of Psychology, University of Waikato, Hamilton, New Zealand; 4 School of Social Sciences, University of Waikato, Hamilton, New Zealand; Manchester Metropolitan University, UNITED KINGDOM

## Abstract

In this paper, we evaluate the impacts of one-way door and CitySafe patrol policies in Whangarei, New Zealand, using a mixed-methods approach. In the quantitative analyses, we apply interrupted time series analysis and difference-in-differences analysis to data on antisocial behaviour derived from CCTV footage, and to police calls-for-service data. In the qualitative analysis, we apply thematic analysis to data from semi-structured interviews with 33 local stakeholders. We find a statistically significant increase in observed antisocial behaviour, but statistically significant decreases in violence and drug and alcohol offences, except when other small cities are used as a control group. In the qualitative analysis, a large majority of interviewees thought that the policy had reduced alcohol-related harm and increased safety, although a number of possible unintended consequences were also noted, including a reallocation of police resources, a redistribution of night-time drinking towards the suburbs, and a change in the demand for taxi companies. Overall, there is evidence only that the policies have reduced perceived alcohol-related harm, rather than reducing measures of harm.

## Introduction

A one-way door (or lockout) restriction is a policy whereby a bar (or other on-licence outlet) may allow patrons to leave the premises but not to re-enter once they have left. The restriction is often (but not always) employed uniformly across all premises in a given entertainment district, and is intended to reduce alcohol-related harm occurring in the night-time economy [1]. However, it is by no means certain that reductions in harm would be the outcome of such a policy, with extant theory consistent with both decreases and increases in harm.

Routine activity theory [2, 3] postulates that violence and other crime may occur when motivated offenders interact with potential victims, in the absence of a suitable guardian. In such interactions, alcohol is a ‘chemical facilitator’ of crime [4], releasing offenders from moral constraints and inhibitions about risk, as well as impairing the potential victim. Routine activity theory [5] suggests that a one-way door restriction could have multiple effects on alcohol-related harms. On the one hand, the movement of people between bars and nightclubs during the night increases the likelihood of intoxicated people interacting [6], and a greater volume of interactions is associated with greater violence and harm [7]. A one-way door restriction reduces this ‘churn’ of patrons moving between licensed premises, and thereby reduces the potential for conflict or victimization [8]. In effect, patrons may stay in a single venue rather than moving between them. On the other hand, a universal closing time for all bars within an entertainment precinct leads to large numbers of people leaving bars and nightclubs simultaneously. This creates a target-rich environment and increases the number of interactions between alcohol-impaired people [9]. A one-way door restriction may concentrate the departure times of patrons into the latter part of the evening, leading to more interactions between patrons exiting licensed premises. In particular, large numbers of people may seek transport to their homes at the same time, creating a potential problem of excess demand and conflict over transport [10]. Moreover, a one-way door restriction may increase conflict between security staff and impaired patrons seeking to enter a licensed premises after the one-way door becomes active.

The theoretical impact of a one-way door restriction on alcohol-related harm is therefore ambiguous, and the net effect is an empirical question. However, as noted below, the extant empirical literature is also inconclusive, often because other concurrent policy changes confound the analysis [11]. In this paper, we report on a mixed-methods evaluation of a one-way door and CitySafe patrol policy implemented in the small city of Whangarei (population approximately 92,000) in the North Island of New Zealand. Whangarei District Council implemented a one-way door policy for the Whangarei Central Business District (CBD), with effect from 7 April 2015, wherein all licensed premises were required to have a one-way door restriction in place between 1 a.m. and unchanged closing times at 3 a.m. On the same date that the one-way door restriction was put in place, CitySafe officers (private security contracted by the Whangarei District Council) began regular patrols of the Whangarei CBD. We argue that the one-way door restriction was the key intervention component of the combined policy, and it is the focus of much of our analysis, but in reality both components were acting concurrently.

### Previous literature

Ours is not the first study to evaluate one-way door restrictions. Nepal et al. [1] conducted a systematic review of the literature up to June 2017, and found eight studies that meet their criteria. Of the eight studies, two showed a decline in assaults, one showed a decline but only inside licensed premises, and two showed an increase in assaults. The other three studies showed no effect.

Most of the evaluative research on lockouts has been conducted in Australia. Kypri et al. [12] reported that the introduction of a 1:30 a.m. lockout and 3:30 a.m. closing in Newcastle (NSW) reduced the incidence of assault by 37%. In a subsequent five-year follow-up on the same intervention, Kypri et al. [13] reported that the reduction in assault rates had been sustained. However, they also reported that the same lockout restriction in nearby Hamilton had no effect on assault rates. The Newcastle lockout policy was not a pure lockout intervention, in that a number of other alcohol outlet management strategies were implemented at the same time. Thus, it is difficult to attribute the effect to the lockout itself, or to identify the proportion (if any) of the reported change in assault rates in Newcastle that can be attributable to the lockout policy rather than the other contemporaneous policy changes.

Mazerolle et al. [14] evaluated the impact of 3 a.m. lockout legislation on violence in and around two entertainment districts in Queensland (Australia) and found that the lockout led to a direct and significant reduction in the number of violent incidents inside licensed premises, but no change outside licensed premises. In contrast, De Andrade et al. [15] found that a pilot 3 a.m. lockout policy in Surfers Paradise (Queensland) had no statistically significant impact on rates of crime, violence, head and neck injuries, or intoxication over the two years following its implementation. Moreover, there was limited evidence of displacement of crime to surrounding areas outside of those affected by the lockout. Finally, Menéndez et al. [16] found a 45% reduction in non-domestic assault in Kings Cross and Sydney CBD (NSW) following the January 2014 introduction of a 1:30 a.m. lockout, with no evidence of spillover to surrounding areas. They found that there had been a reduced incidence of assault in the Kings Cross and CBD Entertainment Precincts. However, Hughes and Weedon-Newstead [17] reported qualitative evidence that alcohol-related problems had increased in nearby Newtown.

In New Zealand, a voluntary one-way door restriction was temporarily implemented in central Christchurch from October 2006 to March 2007. This involved a one-way door policy on Thursday, Friday and Saturday nights from 4 a.m. Kirkwood and Parsonage [18] found that the goal of a 10% reduction in alcohol-related crime and violence in the inner city was not met and that crime actually increased, but that there were reductions in some subsets of crime, such as serious violence offences on Saturday nights/Sunday mornings. There was also a positive impact on key stakeholders’ perceptions of safety and crime levels. The majority of licensees reported that their revenue had not been adversely affected by the policy.

## Materials and methods

Given the lack of consistent evidence on one-way door restrictions (or lockouts), we report on an evaluation of the Whangarei one-way door and CitySafe patrol policy. Our evaluation involves both quantitative and qualitative elements, i.e. it is a mixed-methods evaluation. However, we must note a key limitation from the outset. While it would be helpful to evaluate the two components of the policy (one-way door and CitySafe patrols) separately, both were implemented on the same date. Thus, similar to some of the Australian studies and the Christchurch study cited above, it is not possible to evaluate the effect of the one-way door restriction in isolation. This is because, for any observed quantitative impact, we cannot disentangle the effects of the one-way door restriction from the effects of the CitySafe patrols. Under the circumstances, the quantitative evaluation (and aspects of the qualitative evaluation) should be interpreted as being an evaluation of the package of policy (one-way door restrictions, and CitySafe patrols) implemented in April 2015.

The mixed-methods approach is suitable in this context because the aim was to understand the effects of the policy in a more complete manner. A quantitative analysis alone might be used to identify a quantitative impact on outcomes that are measurable, but miss important spillover effects or unintended consequences arising from the policy, as well as impacts that are difficult or impossible to measure. A qualitative analysis alone might be used to understand the perceptions of the policy implementation and its effects ‘on the ground’, while failing to recognise changes in measurable outcomes.

Our mixed-methods design did not *a priori* adopt an explicit theoretical lens. The quantitative and qualitative elements of the research were conducted concurrently, and served slightly different (but aligned) purposes in the overall evaluation of the one-way door and CitySafe policies (following the methods perspective described in [19]). The quantitative analysis, which we describe first, concentrated on evaluating the impact of the policy implementation on observable outcome measures, including antisocial behaviour and police calls-for-service. The qualitative analysis, which we describe second, focused on evaluating the experiences and views of key informants, to evaluate the implementation of the policy, perceptions of its success, and any unintended consequences that arose from the implementation. Thus, the quantitative and qualitative elements of the study complemented each other, extending the breadth of the research beyond what would have been possible with one method alone [20]. The key advantage of adopting this approach is that it ensured a more holistic evaluation of the effects of the policy change.

The qualitative research received ethics approval from the University of Waikato Human Research Ethics Committee. The quantitative research was exempted from specific ethics approval, due to its exclusive use of secondary and anonymised administrative data.

### Quantitative analysis

In the quantitative data analysis, we adopted a quasi-experimental approach. This essentially involves a comparison of the number of events (observed incidents of antisocial behaviour, or police calls-for-service) occurring in the period before the introduction of the policy was implemented, with the number of such events occurring in the period after the policy was implemented. We used two data sources for the quantitative analysis: (1) observational data on antisocial behaviour from Whangarei District Council; and (2) police calls-for-service data from the New Zealand Police.

Antisocial behaviour data covering the period from 4 November 2011 to 30 October 2016 were derived from Whangarei District Council’s network of CCTV cameras located throughout the CBD. Volunteer observers employed by the Council routinely code incidents observed on the CCTV footage into fifteen categories, of which six are the focus of our analysis: Breach Liquor Ban, Damage, Disorder, Drugs, Intoxicated, and Gangs. Counts of incidents were aggregated to produce a single measure of antisocial behaviour for each weekend (comprised of 6 p.m. Friday to 6 a.m. Saturday, and 6 p.m. Saturday to 6 a.m. Sunday).

We applied an interrupted time series analysis [21], essentially evaluating whether there were level or trend changes in the counts of antisocial behaviour before and after the implementation of the policy change. We utilise a Poisson-based methodology that deals with count, autocorrelation and dispersion issues within a single framework [22], and which has been used extensively in the analysis of count time series (see also [23]). The analysis controls for a separate linear time trend for the period before, and the period after, the implementation date. An alternative model, accounting for up to four lags in the counts of antisocial behaviour was trialled, in order to pick up nonlinearities in the relationship over time, but that specification did not improve the fit (results are available from the authors on request; see also S1 Fig in the Supplementary Materials).

Data on police calls-for-service were obtained from the New Zealand Police Communications and Resource Deployment (CARD) database, covering the period from 6 January 2006 to 26 March 2017. Following Cameron et al. [24], the data were cleaned and restricted to events that were coded to specific offences. We further restricted our analysis to categories that were most likely to relate to alcohol-related offending (Violent offences; Property damage; and Drug and alcohol offences), as well as total calls-for-service. The data were geo-coded using an automated process in ArcGIS, then converted to counts of each category (and total calls-for-service) per weekend for the Whangarei CBD area. We used the same method to derive counts per weekend for the Whangarei suburban area (i.e. excluding the CBD), and for the CBDs of three similarly sized regional cities in the North Island of New Zealand—Rotorua (population 73,000), Gisborne (50,000), and Whanganui (45,000). The CBD areas of these other cities were determined by visual inspection of the pattern of police events, while the area of Whangarei outside the CBD was defined by the overall settlement pattern shown on Google Maps.

To analyse the police calls-for-service data, we first applied the interrupted time series analysis for the Whangarei CBD alone, as described above for antisocial behaviour. We then applied a difference-in-differences (DID) approach (see [25]), using two alternative control groups: (1) the area of suburban Whangarei, where the one-way door and CitySafe patrol policy was not in force; and (2) the average of the CBD areas of Rotorua, Gisborne, and Whanganui. Using suburban Whangarei as a control allows us to control for locally-specific common trends in calls-for-service over time, as well as considering any displacement effects of the policy to other parts of the city. If there are significant displacement effects, then the estimated impact of the policy would be larger (a greater reduction in calls-for-service would be observed for the treatment area, compared with the control area). Using the average of three other cities as a control (rather than a single city) reduces the amount of random noise observed in the control group, which is important given the small counts of some events (e.g. violence) each weekend. In both cases (interrupted time series and DID), we apply negative binomial regression, which adequately deals with the observed over-dispersion of the calls-for-service data. All quantitative analyses were conducted with Stata v15.

Summary statistics on all of the variables used in the quantitative analysis are provided in Table 1, disaggregated into the period before, and the period after, implementation date (7 April 2015). The mean number of daily antisocial behaviour events is substantially higher after implementation than before, while for the police calls-for-service (both for the specific types considered, and in total), the mean number of daily events is lower after implementation than before. However, a simple comparison of these means does not account for underlying time trends in the data. For the police calls-for-service data, the average of the three control cities has a similar distribution of events to the Whangarei CBD, while suburban Whangarei (excluding the CBD) has a greater number of events (with the exception of drug and alcohol offences, where the distributions are similar). This suggests that the average of the three control cities may provide a more appropriate counterfactual than suburban Whangarei, due to the difference in scale, alongside the difference in context (suburban vs. central business district).

**Table 1:** Summary statistics.

	Before implementation					After implementation				
	Mean	Median	SD	Min	Max	Mean	Median	SD	Min	Max
*Antisocial behaviour*										
Whangarei CBD	4.10	3.00	3.20	0.00	18.00	9.80	9.50	5.40	0.00	29.00
*Violent offences*										
Whangarei CBD	0.58	0.00	0.80	0.00	4.00	0.32	0.00	0.55	0.00	2.00
Suburban Whangarei	1.95	2.00	1.43	0.00	7.00	1.73	2.00	1.60	0.00	8.00
Control cities (average)	0.50	0.00	0.72	0.00	4.00	0.35	0.00	0.62	0.00	3.00
*Property damage*										
Whangarei CBD	0.37	0.00	0.60	0.00	3.00	0.21	0.00	0.50	0.00	2.00
Suburban Whangarei	1.54	1.00	1.41	0.00	8.00	0.84	1.00	0.90	0.00	4.00
Control cities (average)	0.31	0.00	0.58	0.00	4.00	0.26	0.00	0.56	0.00	3.00
*Drug and alcohol offences*										
Whangarei CBD	0.58	0.00	0.96	0.00	6.00	0.20	0.00	0.49	0.00	2.00
Suburban Whangarei	0.21	0.00	0.50	0.00	3.00	0.20	0.00	0.40	0.00	1.00
Control cities (average)	0.53	0.00	1.19	0.00	13.00	0.21	0.00	0.56	0.00	5.00
*Total calls-for-service*										
Whangarei CBD	5.08	5.00	2.93	0.00	20.00	3.97	4.00	2.43	0.00	12.00
Suburban Whangarei	16.52	16.00	5.59	3.00	39.00	14.85	14.00	4.11	7.00	26.00
Control cities (average)	4.82	4.00	3.08	0.00	23.00	3.66	3.00	2.50	0.00	21.00

N.B. Interrupted time series analysis evaluating the impact of the introduction of the one-way door and CitySafe patrol policy. Results are reported as incidence-rate ratios, with standard errors in parentheses;

*** *p*<0.01;

** *p*<0.05;

* *p*<0.1.

### Qualitative analysis

Alongside the quantitative analysis, we collected qualitative information via semi-structured interviews with stakeholders in November 2016, in order to contextualise the implementation of the policy. There were a number of research aims for the qualitative research. First, we wanted to know if the one-way door policy was being implemented as intended and the nature of any variations from the stated policy. Second, we wanted to know what impacts, if any, the one-way door and CitySafe patrol policies may have had. While we were mostly interested in impacts on alcohol-related harm and perceptions of safety in the CBD, we were also interested in perceived impacts on the wider city, including possible unintended impacts (e.g. displacement of harm, or perceived impacts on the night-time economy). Finally, we were interested in what, if any, other changes had occurred in the city that may have had an impact on the level of alcohol related harm and perceptions of safety. That is, we wanted to know if participants could identify any possible confounding factors, as well as any unintended consequences that may have arisen.

Participants were recruited primarily through purposive sampling. Many of the relevant stakeholders had been publicly involved in the formative stage of the one-way door policy. Other participants were identified through recommendations by the implementing agency (Whangarei District Council) or though an initial stakeholder meeting at the commencement of the research. In total, we interviewed 33 people from eight main stakeholder groups, including those with a statutory responsibility for licensing matters (Whangarei District Council, New Zealand Police; *n* = 9) or for managing alcohol-related health issues (Northland District Health Board; *n* = 2), businesses that are likely to be affected by the policy (central city bars, taxi companies, and other businesses that operate in the night-time economy; *n* = 17), and others with a role in enhancing the safety of the inner city (CitySafe, community patrols, and the volunteers who monitor the CBD CCTV cameras; *n* = 5). An additional five people were contacted, but were not interviewed for various reasons (declined, new to their role, too busy). However, despite the unavailability of those potential participants, the remaining stakeholders represent an adequate coverage of the relevant stakeholder groups.

It should be noted that, to varying degrees, our participants had “skin in the game.” Although most participants had been involved in the development of the one-way door policy, their different positions (particularly policy or harm reduction vs. business) were likely to result in their having varying views about its value, its likely efficacy and its desirability, as assessed from their own perspective. Moreover, the participants varied in the extent to which they had line-of-sight on the implementation and impacts of the policy. That is, some had a hands-on role in the hospitality industry and/or were in a position to view first-hand at least the some of anti-social behaviour we were interested in, while others were more distant observers, often relying on second-hand information from a variety of sources. It was important for us to reflect on these positional differences as we recruited participants, as we conversed with them and as we weighed up what they told us.

We also needed to be mindful of our own positionality as researchers. While we undoubtedly placed value on public safety, we were agnostic on whether a one-way door policy was an effective way of enhancing safety in the context of the night-time economy. That is, while we reject the notion that we could enter research *tabula rasa*, we did see ourselves as neutral on the questions we set out to address.

All interviews were digitally recorded, and selectively transcribed. We then utilised deductive thematic analysis [26]. First, high level themes were identified that mapped on to the key issues we wanted to address: fidelity of implementation; relevant changes in the setting; the impact of the policy on alcohol-related harm in the Whangarei CBD; unintended consequences of the policy; possible refinements to the policy; and process factors relating to the development and implementation of the policy. Second, we categorised sections of the text under one or more of these themes. Third, within these high-level themes, we identified sub-themes—particular patterns among the responses that provided various accounts of the main themes. In evaluating the responses, we prioritised those from interviewees who were in the best position to comment on particular issues, generally because they were reporting on things that they had observed directly rather than reporting things that they had heard from others.

## Results

### Quantitative analysis

Table 2 summarises the results of the interrupted time series analysis (as incidence-rate ratios), both for antisocial behaviour and police calls-for-service (in total and by category). These results are summarised in graphical form in the Supplementary Materials, S1–S5 Figs. For antisocial behaviour, the only statistically significant variable is the post-intervention dummy variable, with an incidence-rate ratio of more than three. This indicates that there was a statistically significant increase in the number of antisocial behaviour incidents recorded from the CCTV data between the period before the implementation of the policy and the period after. Specifically, the incidence rate was three times higher after the policy was implemented. In contrast, when police calls-for-service is the dependent variable, the post-intervention dummy variable is statistically significant for both violent offences and drug and alcohol offences, in both cases with an incidence-rate ratio of less than one, i.e. a statistically significant decrease in calls-for service for those categories.

**Table 2:** Interrupted time series analysis results.

Variable	Antisocial behaviour	Violent offences	Property damage	Drug and alcohol offences	Total calls-for-service
*T* (time since implementation of the policy)	0.998 (-0.001)	0.999** (<0.001)	1.000 (0.001)	1.001* (0.001)	1.000 (<0.001)
*X*_*t*_ (post-intervention dummy variable)	3.312*** (0.166)	0.355** (0.157)	0.773 (0.332)	0.336** (0.166)	0.837 (0.107)
*TX*_*t*_	0.997 (-0.003)	1.014** (0.006)	0.995 (0.007)	0.995 (0.008)	1.000 (0.002)
*N*	261	570	570	570	570
*Adjusted R* ^*2*^	0.309	0.018	0.007	0.020	0.004

N.B. Interrupted time series analysis evaluating the impact of the introduction of the one-way door and CitySafe patrol policy. Results are reported as incidence-rate ratios, with standard errors in parentheses;

*** *p*<0.01;

** *p*<0.05;

* *p*<0.1.

Table 3 presents the results of the DID analysis, using suburban Whangarei as a control (Panel A), and the average count of calls-for-service for Rotorua CBD, Gisborne CBD, and Whanganui CBD combined as a control (Panel B). In Panel A, the variable of interest is statistically significant with an incidence-rate ratio of less than one for violent offences, drug and alcohol offences, and total calls-for-service. This demonstrates that calls-for-service declined in Whangarei CBD at a faster rate after the policy was implemented, compared with the rest of Whangarei. Specifically, the incidence rates were 38 percent, 64 percent, and 13 percent lower for violent offences, drug and alcohol offences, and total calls-for-service respectively. However, suburban Whangarei might not be the most appropriate counterfactual for changes in the Whangarei CBD, due to a difference in context, and the potential for spillover effects from the CBD to the rest of the city. In the bottom panel of Table 3, with our preferred counterfactual of the average of three other city CBDs, the variable of interest is statistically insignificant in all models. This suggests that, relative to calls-for-service in the CBDs of other regional cities, the calls-for-service in Whangarei CBD did not change after the policy was implemented.

**Table 3:** DID analysis results.

Variable	Violent offences	Property damage	Drug and alcohol offences	Total calls-for-service
***Panel A*** *				
*D* (post-intervention dummy variable)	0.885 (0.077)	0.547*** (0.066)	0.975 (0.255)	0.899*** (0.035)
*M*_*t*_ (treatment dummy variable)	0.299*** (0.021)	0.237*** (0.021)	2.792*** (0.367)	0.308*** (0.009)
*DM*_*t*_ (DID variable of interest)	0.620** (0.127)	1.066 (0.278)	0.358*** (0.129)	0.869** (0.062)
*N*	1172	1172	1172	1172
*Pseudo R* ^*2*^	0.110	0.114	0.042	0.165
***Panel B*** ^†^				
*D* (post-intervention dummy variable)	0.692*** (0.076)	0.824 (0.105)	0.394*** (0.068)	0.760*** (0.034)
*M*_*t*_ (treatment dummy variable)	0.389*** (0.028)	0.389*** (0.036)	0.370*** (0.036)	0.351*** (0.010)
*DM*_*t*_ (DID variable of interest)	0.793 (0.172)	0.707 (0.185)	0.887 (0.272)	1.029 (0.079)
*N*	1172	1172	1172	1172
*Pseudo R* ^*2*^	0.070	0.059	0.051	0.133

N.B. Difference-in-differences (DID) analysis evaluating the impact of the introduction of the one-way door and CitySafe patrol policy. Results are reported as incidence-rate ratios, with standard errors in parentheses;

*** *p*<0.01;

** *p*<0.05;

* *p*<0.1;

* Panel A reports results using suburban Whangarei (excluding the CBD) as a control group;

^†^ Panel B reports results using the average of the CBD areas of Rotorua, Gisborne, and Whanganui as a control group.

### Qualitative analysis

A number of high-level themes were identified in the qualitative research, including: fidelity of implementation; relevant changes in the setting; the impact of the policy on alcohol-related harm in the Whangarei CBD; unintended consequences of the policy; possible refinements to the policy; and process factors relating to the development and implementation of the policy. Each of the following sub-sections addresses one of those themes.

#### Fidelity of implementation

Overall, there was a strong consensus among stakeholders that the policy had been implemented as intended. There was only one incident that was mentioned by several participants, in which the policy appeared to be blatantly breached. It was described by one interviewee in the following terms:

From staff and doorman—what we all observed on the footage is that they were letting people in through the back door and it looked to us as if the doorman was telling people ‘just go round the corner, out the back, and we’ll let you in’. That was nipped in the bud then after we spoke to the licensee, although he didn’t really acknowledge that they were let in.

(Whangarei District Council [WDC]).

Some other “minor slippages” were observed: “occasional” cases of someone being allowed back into a bar, and some laxity about timing (“five minutes here or there (but), you’ve got to provide for a margin of error”). Such departures from “good practice” were routinely followed up. As was pointed out by several participants, the availability of CCTV footage, the presence of CitySafe officers and the fact that Whangarei is “a pretty small town (in which) we talk a bit between ourselves” all meant that the policy was closely monitored and corrective action was taken when needed.

#### Relevant changes in the setting

Our participants identified a number of changes in the setting that might have had an impact on night-time safety in the inner city. First, communication between CitySafe officers, the CCTV volunteers, and the police is believed to have improved the police response to incidents in the CBD, as explained by a council officer:

You know what we have seen has been improved responses from the police. So initially we were challenged by some response times by the police, but with the one-way door policy, what we have drawn down from that, is that the timeframe for incidents occurring has narrowed quite a bit, and that’s assisted police with resource deployment. And in fact what we’ve got from that is better response times from the police. So it’s great to have a CCTV system, but when we observed something happening it’s there to enable the police to get there quicker and to be able to know what they’re dealing with, where they’re dealing with it, and when. So they can get there faster.

(WDC).

Here, the description of an improved police response is given by someone outside the police who had previously been critical of the police. We think this adds to its credibility. It also raises an interesting issue about the evaluation of one-way door policies (and probably other initiatives involving community collaboration). That is, where a one-way door policy is found to have decreased anti-social behaviour, it is possible that at least some of the reduction is attributable not to the policy per se, but to the improved cooperation between community partners through the development and implementation of the policy. This reinforces the need for careful formative and process evaluation to sit alongside outcome evaluation. To rely solely on outcome evaluation may give rise to findings which are best simplistic, and at worst, misleading [27].

Second, CitySafe Officers have been on patrol since the implementation of the policy, with four officers originally being employed to work on weekends from 12 a.m. to 4 a.m. It seems plausible to assume that both the presence of CitySafe officers and a quicker police response would enhance safety in the CBD, reducing alcohol-related harms.

Third, a number of changes to the operation of licensed premises within the CBD setting were also noted. According to a licensee, “Pretty much every big hospitality business in the CBD has changed hands” since the policy was put in place. Several licensed premises within the CBD closed after the implementation of the one-way door restrictions, including the largest nightclub in the CBD. Some participants commented that the closure of “bad” or “unsavoury” bars can lead to a significant improvement in the behaviour that occurs within the CBD:

Now that [bar] is gone completely… the whole town has changed. Completely. There’s no cars cruising round with attitude, there’s no people from that bar with attitude, they’ve all gone home or gone somewhere else, and last weekend we’ve had one of the quietest weekends that we’ve ever had, and that is purely because that bar has closed

(CCTV volunteer).

Potentially, each of the changes mentioned above may have contributed to a reduction of alcohol-related harm in the CBD. In terms of evaluating the outcome of one-way door restrictions, these changes are plausible confounding factors.

#### The impacts on alcohol-related harm

When asked about changes that they had observed in alcohol-related harm and crime, a large majority of interviewees perceived that the policy had reduced alcohol-related harm and that the CBD was now safer at night. Importantly, Police participants were unanimously of the view that the policy had improved the situation. For example:

I personally, from what I’ve seen, I think there’s been a huge reduction in disorder and crime in town, purely because I don’t think you get all the hangers-on sort of hanging around outside the clubs and things. They know at one o’clock they can’t get in, so it’s just time to go home. Whereas prior to that policy, you know you’d just have dozens of people just sitting on the side of curbs and footpaths and hanging outside clubs because they’ve got nothing else to do.

(Police Officer).

The positive accounts from police officers were corroborated by a member of a different group of stakeholders—a licensee:

I’ve noticed big changes for us in a positive way, for example from 1 a.m.–because as an owner I’m actually here every weekend so I see it 100% everything that happens, I work on the door—from 1 a.m. my stress level has gone from 100% down to probably 30% due to the simple fact that we don’t have to worry about what’s walking up the street, you know, we can concentrate on what we’re doing… Aggression at the door has decreased, I would say 90%…. due to the fact that we don’t have to say to people “Hey sorry mate you can’t come in, you’re too intoxicated” or “you’ve got the wrong shoes” or whatever reason. Quite simply we say “Sorry guys, it’s against the law and monitored by the police. There’s nothing we can do.” So in that respect it has been very positive

(Licensee).

Most of the other interviewees who were in a position to observe early morning behaviour on inner city streets also gave positive accounts of the impact of the policy on alcohol-related harm and safety. Both taxi drivers we interviewed considered that the streets were quieter and that there were fewer intoxicated people about. Similarly, one of the late-night food vendors we interviewed commented that the CBD is “a safer place now because there’s less people there.”

However, some interviewees were not so positive. For example, one of the volunteers monitoring inner city security cameras thought that there had been a reduction in alcohol-related harm only to “some degree” and that it was “hard to gauge.” Similarly, when asked if the CBD was safer as a result of the policy, one of the late-night food vendors replied:

No not really, because I’ve been told of people waiting in doorways for people that have been mugged or smacked and tried to grab their wallet and things like that. So I’m still hearing a lot of that.

(Late night food vendor).

Scepticism about the effectiveness of the policy was strongest among interviewees involved in the hospitality industry. Within this group, the majority thought that the policy had made little or no difference to alcohol-related harm or inner-city safety. Nevertheless, there was some ambiguity in such responses. For example, one licensee initially said he had not noticed any reduction in problem behaviour because:

At the end of the day, at 3 o’clock in the morning, there’s still a whole lot of drunk people released on the streets.

(Licensee).

He went on to say that there was more drinking in the streets by people who cannot get into bars after the lockout, and that much of the trouble was caused by people too young to get into bars and thus unaffected by the policy. However, later in the interview he commented:

Yes probably there has been a reduction in crime but that’s because there’s a reduction of people physically out (on the streets).

(Licensee).

#### Unintended consequences

While the aim of the policy was to reduce alcohol-related harm and crime in the Whangarei CBD, it may also result in other (unintended) consequences. In this category, respondents noted: (1) a reallocation of police resources towards crime prevention and response outside of the CBD; (2) fewer people coming into town at night; (3) people who might previously have come into the CBD are now drinking at private parties and public venues in the suburbs; and (4) a change in the pattern of business for taxi companies. Naturally, the latter three of these effects are closely related. One taxi driver commented:

We’re carrying a lot more people to the liquor shops and that before half past ten-ten o’clock to pick up booze, and take home. It’s stopped a lot of people coming into town to drink; they’re now drinking at home.

(Taxi driver).

#### Possible refinements to the policy

Some participants suggested ways in which the one-way door policy could be modified to improve its effectiveness. The main suggestion was that the policy allow for some flexibility. That is, patrons who had a good reason to go outside for a time could be given a ‘pass-out’ card that would allow them to re-enter the premises. This could be used, interviewees said, by a sober driver who could take a friend home and then return for others, or to escort a woman to a taxi or to her parked car.

#### Process factors

Finally, we asked participants to reflect on the way various organisations worked together on the development and implementation of the policy. In this respect, while interviewees acknowledged that there had initially been some operational factors to iron out, they were generally of the view that there is a strong relationship between CitySafe officers, police, Whangarei District Council, and the CCTV volunteers. The positive communication between these groups means that they have been able to implement the policy effectively and successfully.

## Discussion

Our quantitative analysis of the policy changes to introduce a one-way door restriction and CitySafe patrols in Whangarei in 2015 demonstrated a statistically significant increase in antisocial behaviour observed in CCTV footage after the implementation of the policy. The approximate tripling of antisocial behaviour incidents per weekend after implementation of the policy would seem to be perverse given that one of the aims of the policy was to reduce the incidence of antisocial behaviour. There are a number of possible explanations of this result. The most plausible explanation centres on the interaction between CitySafe patrols and data collection. Our qualitative analysis revealed close relationships between CitySafe, police, and the CCTV volunteers. In particular, CitySafe officers directed the CCTV cameras to particular events, or asked if the volunteers had observed particular events. It would seem reasonable then to argue that the observed increase in instances of antisocial behaviour was not due to the policy itself, but was rather due to the interaction between CitySafe and the CCTV volunteers. In other words, the policy may not have led to an increase in antisocial behaviour in the Whangarei CBD, but instead to an increase in the number of recorded events due to greater vigilance in the monitoring of the CBD.

In contrast, an evaluation of Police calls-for-service data suggested that there had been statistically significant decreases in violent offences and drug and alcohol offences in the Whangarei CBD. Specifically, the ratios (0.355 for violent offences, and 0.336 for drug and alcohol offences) suggest that there was a decrease by around two-thirds in these events after the policy was implemented. However, there was no significant effect on property damage or on total calls-for-service. The latter is somewhat surprising, since it suggests that, while there may have been decreases in violent offences and drug and alcohol offences, these decreases were offset by increases in calls-for-service for other types of crime in the Whangarei CBD. Moreover, the decrease in violent offences appears to have been countered somewhat by an increasing trend since the implementation of the policy, such that the number of violent offences had returned to its long-run trend by March 2017.

Further analysis of the police calls-for-service data using a difference-in-difference approach revealed decreases in violent offences, drug and alcohol offences, and total calls-for-service in Whangarei CBD compared with suburban Whangarei, but no decreases relative to the CBD areas of three other regional cities (Rotorua, Gisborne, and Whanganui). However, the DID analysis for Whangarei alone cannot be taken as evidence for the success of the policy. This is because these results could have arisen because of a relative decrease in calls-for-service in the Whangarei CBD, a relative increase in calls-for-service in suburban Whangarei (such as a negative displacement effect of the policy into the rest of the city), or a combination of the two. Nevertheless, combining these results with the earlier reported results from the interrupted time series analysis suggests that much of the effect may be driven by a decrease in calls-for-service in the Whangarei CBD, rather than negative displacement effects.

The DID analysis using the other cities as a control group contrasts strongly with the results of the interrupted time series analysis, and the DID analysis using suburban Whangarei as a control. This may have arisen because the overall time trend in police calls-for-service has been downward in all of these places. If decreases in calls-for-service over time have generally been greater for CBD areas than for suburban areas, then this would explain most of our quantitative results. This would be especially true if time trends diverged close to or after the implementation of the policy. In that case, an interrupted time series analysis would show a decrease in calls-for-service between the period before and the period after the intervention (in part because of the divergence in overall trends in calls-for-service), a DID using suburban Whangarei as a control would show a large impact of the intervention (because of the different trends in calls-for-service between the CBD and the suburban area), and a DID analysis using other city CBDs as a control would show no statistically significant effects (because the observed decline in calls-for-service is a feature of CBDs generally, rather than specific to Whangarei CBD). Overall, this suggests some caution in concluding that there has been a significant quantitative effect of the policy on alcohol-related harm.

The qualitative analysis found that there was a strong consensus that the policy had been mostly implemented as intended. This reflects a high degree of cooperation between the stakeholders. Moreover, the strong relationship between CitySafe officers, police, Whangarei District Council, and the CCTV volunteers and improved communication between these groups was cited as a key factor in the perceived success of the implementation. Building a strong stakeholder commitment to a policy such as this, and supporting that commitment with close partnerships between key actors in the night-time economy, is a lesson that other implementing agencies could draw from this research, not just in relation to one-way door and safety policies, but also more broadly in the area of reducing alcohol-related harms.

Participants noted a number of positive changes in the downtown Whangarei night-time economy. Some of these, including improved communication between CitySafe officers, CCTV volunteers, and Police, present challenging confounders for a ‘pure’ quantitative evaluation of the impacts of the policy on measures of alcohol-related harms (as noted earlier in the Discussion). In contrast, the closure of problematic bars may be seen as a positive and anticipated outcome contributing to reduced alcohol-related harms.

A large majority of interviewees held positive views about the policy, and thought that the combined policy had reduced alcohol-related harm, resulting in a CBD that was now safer at night. This conclusion should be treated with some caution, as it may be subject to some degree of confirmation bias, and we cannot dismiss the possibility that positive changes might be at least partly attributed to confounding factors. Moreover, the *perceived* positive impacts of the policy were not reflected in the quantitative analysis, as noted above.

A number of possible unintended consequences were also noted in the qualitative research, including a reallocation of police resources, fewer people in the night-time economy in Whangarei, and displacement of drinking and alcohol-related harm from the CBD to the suburbs. Unintended consequences are somewhat unavoidable. However, the observed changes noted by our participants should be considered by other implementing agencies seeking to introduce similar policies. Furthermore, our participants noted that the policy could be improved. Refinements of the policy, such as the use of ‘pass-out’ cards, could be considered for the future.

In addition to the limitations noted earlier, our research design, wherein the quantitative and qualitative elements were conducted concurrently, may have somewhat limited the possibilities for a deeper understanding of why the policy implementation did not have its intended effects of reducing alcohol-related harm. Had we undertaken the quantitative analysis in advance of the key informant interviews (in a sequential explanatory design [28]), this may have pointed to additional questions related to impact that could have been explored further. However, delaying the qualitative data collection could have led to biases in the perceived impacts of the policy by key informants due to recall bias. Nevertheless, our mixed-methods design allowed us to identify why measured anti-social behaviour increased in the CBD following the implementation of the policy, a key point that might otherwise have been missed. It also tempers the positive impressions of the research participants about the impacts of the policy, which are not wholly supported by the results of the quantitative analysis.

## Conclusion

Overall, we cannot definitively conclude from the quantitative analysis that the Whangarei policy has decreased police calls-for-service or observed antisocial behaviour in the Whangarei CBD. However, based on the qualitative observations of those who are likely to be best able to evaluate the on-the-ground reality, there is evidence only that the one-way door restrictions and CitySafe patrols have reduced perceived alcohol-related harm, rather than reducing measures of such harm. Thus, a CBD safety and one-way door policy may not be sufficient on its own to reduce alcohol-related harm, and consideration of possible unintended consequences is important.

Unfortunately for policy advocates, the inconclusive nature of the quantitative results does not provide strong support for similar policies in other small cities. That does not mean that one-way door policies are necessarily ineffective in all contexts. Future research could add to our understanding of these policies and whether there are particular contexts in which they have noticeable (and measurable) impacts, by evaluating their impact in larger urban centres, or smaller towns, and across areas with a variety of initial levels of alcohol-related harm. Moreover, following up with a further evaluation some years after implementation would enable us to understand the longer-run implications of these policies.

## Supporting information

S1 Fig Antisocial behaviour in Whangarei CBD, 2011–2016—Interrupted time series results. Each point represents one daily observation of the number of antisocial behaviour events in the Whangarei CBD. The vertical line marks the date of implementation of the one-way door and CitySafe policies. The linear trend before and after the date of implementation are illustrated by the red and green trend lines respectively. The red trend line represents the results from an interrupted time series analysis incorporating four lag periods (detailed results are available from the authors on request).
(TIF)

S2 Fig Violent offence calls-for-service in Whangarei CBD, 2006–2017—Interrupted time series results. Each point represents one daily observation of the number of violent offence calls-for-service in the Whangarei CBD. The vertical line marks the date of implementation of the one-way door and CitySafe policies. The linear trend before and after the date of implementation are illustrated by the red and green trend lines respectively.
(TIF)

S3 Fig Property damage calls-for-service in Whangarei CBD, 2006–2017—Interrupted time series results. Each point represents one daily observation of the number of property damage calls-for-service in the Whangarei CBD. The vertical line marks the date of implementation of the one-way door and CitySafe policies. The linear trend before and after the date of implementation are illustrated by the red and green trend lines respectively.
(TIF)

S4 Fig Drug and alcohol offence calls-for-service in Whangarei CBD, 2006–2017—Interrupted time series results. Each point represents one daily observation of the number of drug and alcohol offence calls-for-service in the Whangarei CBD. The vertical line marks the date of implementation of the one-way door and CitySafe policies. The linear trend before and after the date of implementation are illustrated by the red and green trend lines respectively.
(TIF)

S5 Fig Total police calls-for-service in Whangarei CBD, 2006–2017—Interrupted time series results. Each point represents one daily observation of the total number of police calls-for-service in the Whangarei CBD. The vertical line marks the date of implementation of the one-way door and CitySafe policies. The linear trend before and after the date of implementation are illustrated by the red and green trend lines respectively.
(TIF)

## References

[1] Nepal S, Kypri K, Pursey K, Attia J, Chikritzhs T, Miller P. Effectiveness of lockouts in reducing alcohol-related harm: Systematic review. Drug Alcohol Rev. 2018;37:527–536. doi: 10.1111/dar.12699 29675863

[2] Clarke RV, Felson M, editors. Routine activity and rational choice. New Brunswick, NJ: Transaction Publishers; 1993.

[3] Cohen LE, Felson M. Social change and crime rate trends: A routine activity approach. Am Sociol Rev. 1979;44:588–605.

[4] Clarke RV, Eck J. Crime analysis for problem solvers in 60 small steps. Washington, DC: U.S. Department of Justice, Office of Community Oriented Policing Services; 2005.

[5] Clarke RV. Situational crime prevention. Crime Justice. 1995;19:91–150.

[6] Carpenter C, Dobkin C. Alcohol regulation and crime. In: Cook PJ, Ludwig J, McCrary J, editors. Controlling crime: Strategies and tradeoffs. Chicago: University of Chicago Press; 2011. pp. 291–330.

[7] Brantingham PJ, Brantingham PL, editors. Environmental criminology. Beverly Hills, Ca.: Sage; 1981.

[8] Graham K, Homel R. Raising the bar—Preventing aggression in and around bars, pubs and clubs. London: Willan; 2008.

[9] Brantingham P, Brantingham P. Criminality of place. Eur J Crim Pol Res. 1995;3:5–26.

[10] Miller PG, Coomber K, Ferris J, Burn M, Vakidis T, Livingston M, et al. QUeensland Alcohol-related violence and Night Time Economy Monitoring (QUANTEM): Final Report. Geelong, Australia: Deakin University; 2019.

[11] Palk GRM, Davey JD, Freeman J, Morgan H. Perspectives on the effectiveness of late night liquor trading lockout legislative provisions. Crim Justice Policy Rev. 2012;23:465–492.

[12] Kypri K, Jones C, McElduff P, Barker D. Effects of restricting pub closing times on night-time assaults in an Australian city. Addiction. 2011;106:303–310. doi: 10.1111/j.1360-0443.2010.03125.x 20840191 PMC3041930

[13] Kypri K, McElduff P, Miller P. Restrictions in pub closing times and lockouts in Newcastle, Australia five years on. Drug Alcohol Rev. 2014;33:323–326. doi: 10.1111/dar.12123 24589092

[14] Mazerolle L, White G, Ransley J, Ferguson P. Violence in and around entertainment districts: A longitudinal analysis of the impact of late-night lockout legislation. Law Policy. 2012;34:55–79.

[15] De Andrade D, Homel R, Townsley M. Trouble in paradise: The crime and health outcomes of the Surfers Paradise licensed venue lockout. Drug Alcohol Rev. 2016;35:564–572. doi: 10.1111/dar.12384 26913775

[16] Menéndez P, Kypri K, Weatherburn D. The effect of liquor licensing restrictions on assault: A quasi-experimental study in Sydney, Australia. Addiction. 2016;112(2):261–268. doi: 10.1111/add.13621 27658620

[17] Hughes CE, Weedon-Newstead AS. Investigating displacement effects as a result of the Sydney, NSW alcohol lockout legislation. Drugs (Abingdon Engl). 2018;25(5):386–396.

[18] Kirkwood L, Parsonage P. Evaluation of the Christchurch city one-way door intervention—Final report. Wellington: Alcohol Advisory Council of New Zealand and Accident Compensation Corporation; 2008.

[19] Greene JC, Caracelli VJ, Graham WF. Toward a conceptual framework for mixed-method evaluation designs. Educ Eval Policy Anal. 1989;11:255–74.

[20] Creswell JW, Plano Clark VL. Designing and conducting mixed methods research. Thousand Oaks, Ca: Sage; 2007.

[21] Bernal JL, Cummins S, Gasparrini A. Interrupted time series regression for the evaluation of public health interventions: a tutorial. Int J Epidemiol. 2016;dyw098. doi: 10.1093/ije/dyw098 27283160 PMC5407170

[22] Schwartz J, Spix C, Touloumi G, Bachárová L, Barumamdzadeh T, le Tertre A, et al. Methodological issues in studies of air pollution and daily counts of deaths or hospital admissions. J Epidemiol Community Health. 1996;50(Suppl 1):S3–S11. 8758217 10.1136/jech.50.suppl_1.s3 PMC1060881

[23] Katsouyanni K, Schwartz J, Spix C, Touloumi G, Zmirou D, Zanobetti A, et al. Short-term effects of air pollution on health: A European approach using epidemiological time series data. The APHEA protocol. J Epidemiol Community Health. 1996;50(Suppl 1):S12–S18. doi: 10.1136/jech.50.suppl_1.s12 8758218 PMC1060882

[24] Cameron MP, Cochrane W, Gordon C, Livingston M. (2013). The locally-specific impacts of alcohol outlet density in the North Island of New Zealand, 2006–2011. National Institute for Demographic and Economic Analysis, University of Waikato; 2013.

[25] Angrist JD, Pischke JS. Mostly harmless econometrics: An empiricist’s companion. Princeton: Princeton University Press; 2009.

[26] Braun V, Clarke V. Using thematic analysis in psychology. Qual Res Psychol. 2006;3(1):77–101.

[27] Patton MQ, Campbell-Patton CE. Utilization-focused evaluation (5th ed). Saint Paul, Mn.: Sage; 2021.

[28] Ivankova NV, Creswell JW, Stick SL. Using mixed-methods sequential explanatory design: From theory to practice. Field methods. 2006;18:3–20.

