# Supplementary figures and images for: A mixed-methods evaluation of the one-way door and CitySafe patrol policies in Whangarei, New Zealand
Source: PLoS One. 2022 Jun 21;17(6):e0270149. doi: 10.1371/journal.pone.0270149 (PMC9212131; doi:10.1371/journal.pone.0270149)

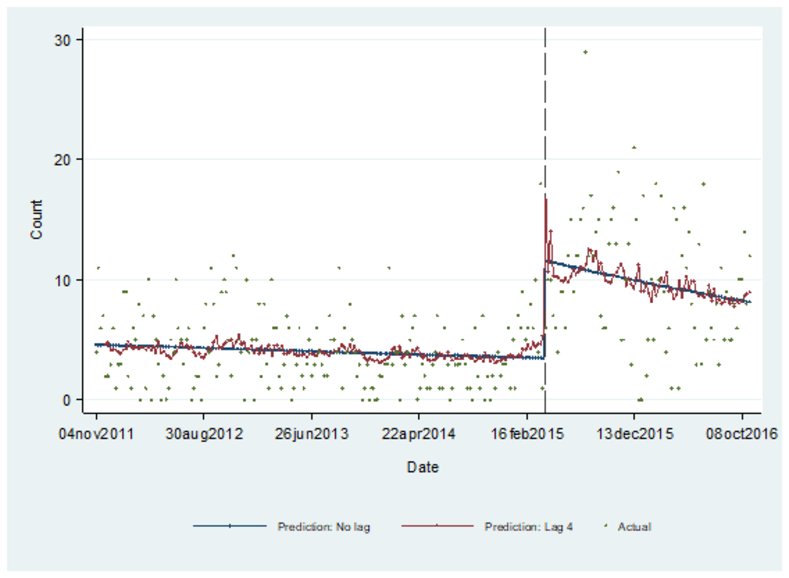

Supplement: S1 Fig — Each point represents one daily observation of the number of antisocial behaviour events in the Whangarei CBD. The vertical line marks the date of implementation of the one-way door and CitySafe policies. The linear trend before and after the date of implementation are illustrated by the red and green trend lines respectively. The red trend line represents the results from an interrupted time series analysis incorporating four lag periods (detailed results are available from the authors on request). (TIF) [file pone.0270149.s001.tif]

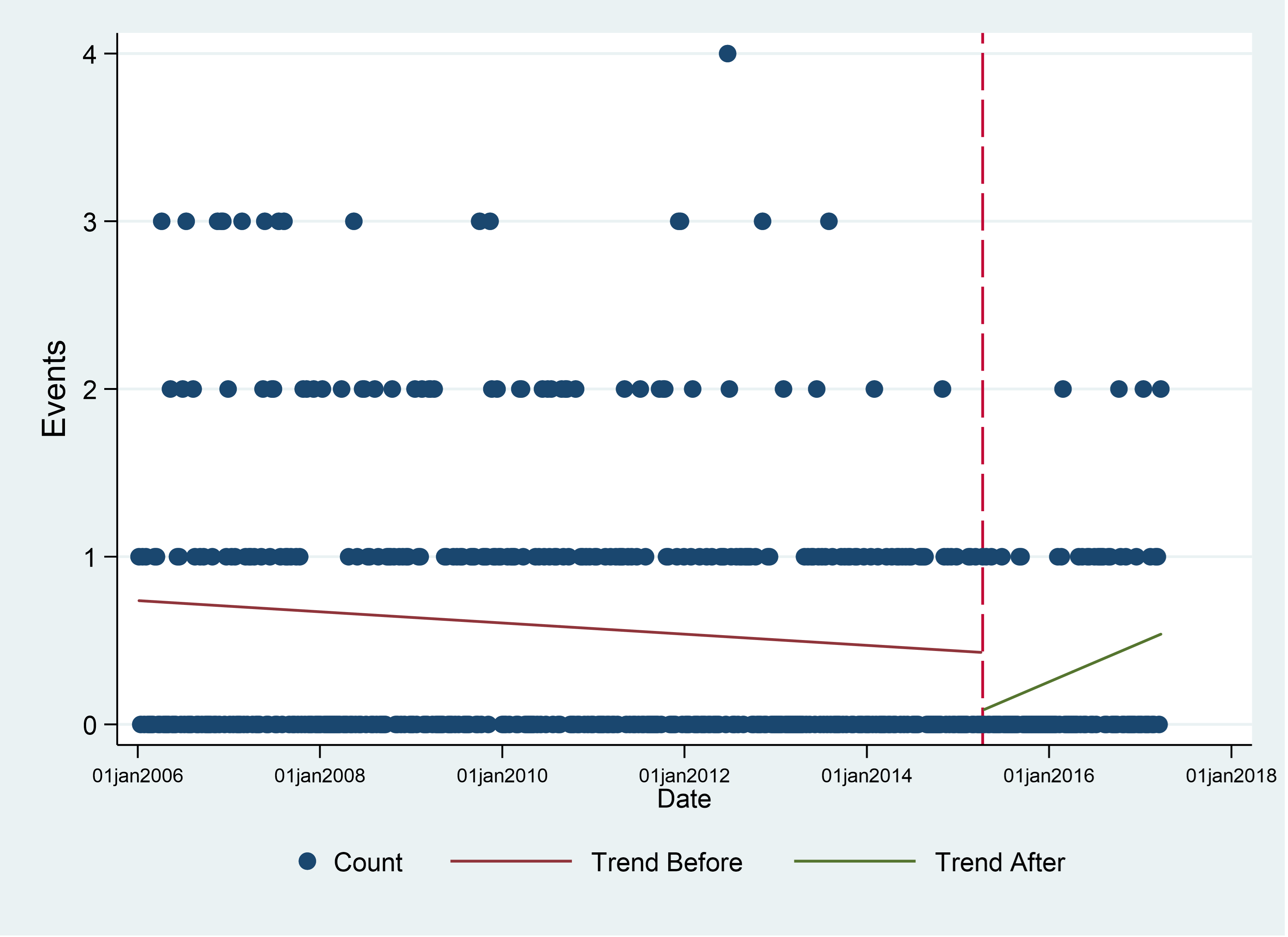

Supplement: S2 Fig — Each point represents one daily observation of the number of violent offence calls-for-service in the Whangarei CBD. The vertical line marks the date of implementation of the one-way door and CitySafe policies. The linear trend before and after the date of implementation are illustrated by the red and green trend lines respectively. (TIF) [file pone.0270149.s002.tif]

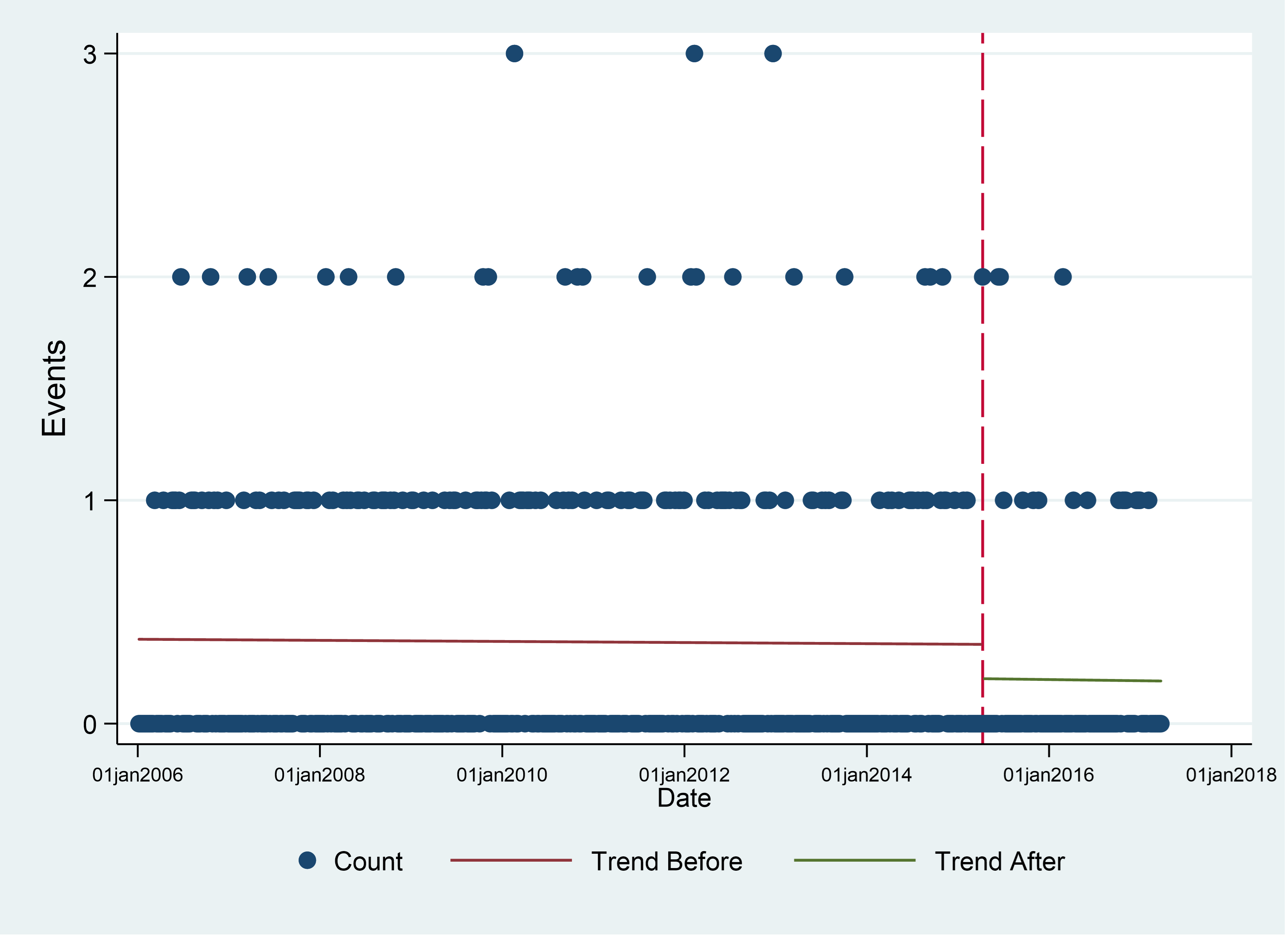

Supplement: S3 Fig — Each point represents one daily observation of the number of property damage calls-for-service in the Whangarei CBD. The vertical line marks the date of implementation of the one-way door and CitySafe policies. The linear trend before and after the date of implementation are illustrated by the red and green trend lines respectively. (TIF) [file pone.0270149.s003.tif]

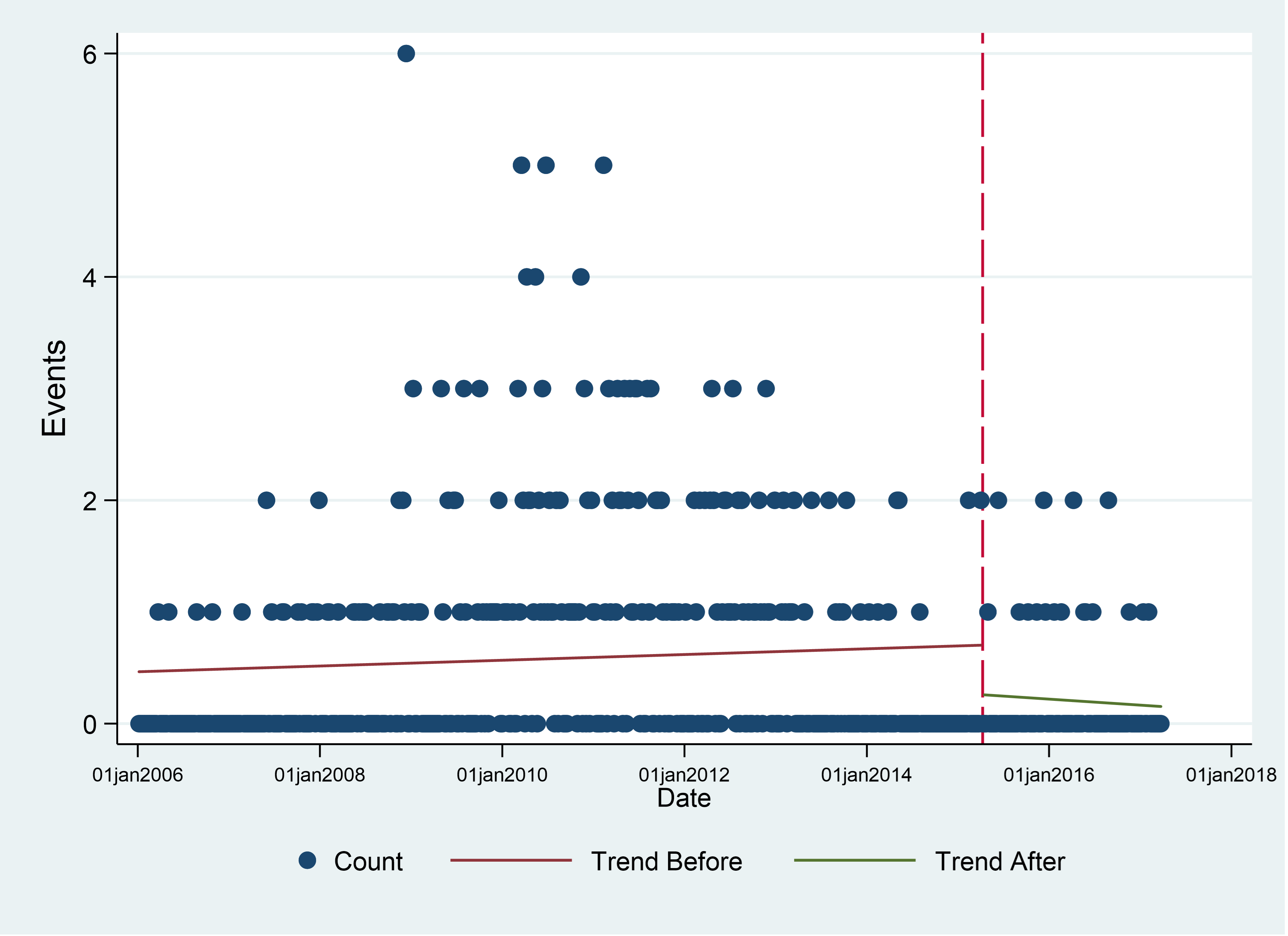

Supplement: S4 Fig — Each point represents one daily observation of the number of drug and alcohol offence calls-for-service in the Whangarei CBD. The vertical line marks the date of implementation of the one-way door and CitySafe policies. The linear trend before and after the date of implementation are illustrated by the red and green trend lines respectively. (TIF) [file pone.0270149.s004.tif]

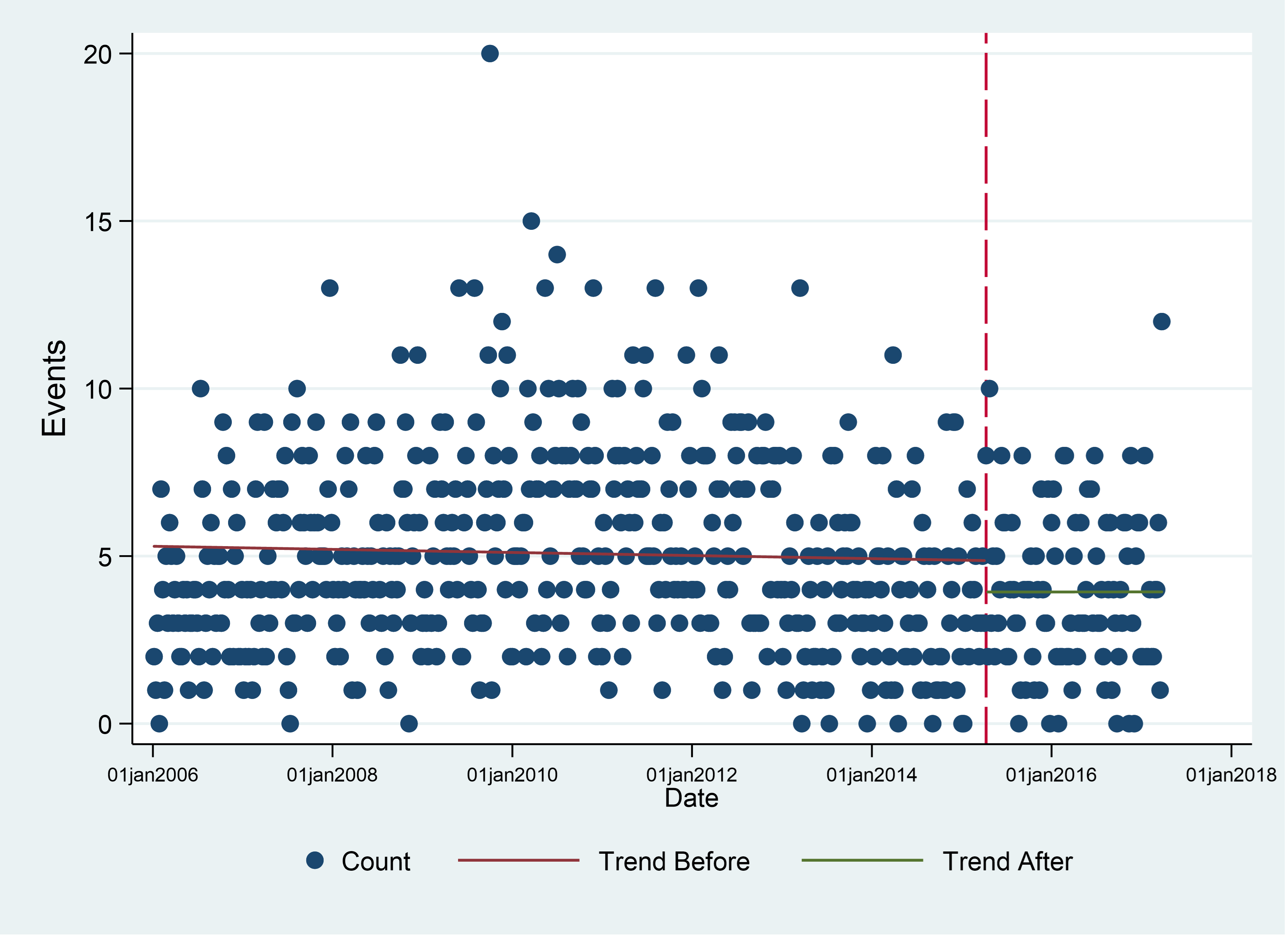

Supplement: S5 Fig — Each point represents one daily observation of the total number of police calls-for-service in the Whangarei CBD. The vertical line marks the date of implementation of the one-way door and CitySafe policies. The linear trend before and after the date of implementation are illustrated by the red and green trend lines respectively. (TIF) [file pone.0270149.s005.tif]
